# Stress About Eviction or Loss of Housing and Child Mental Health

**DOI:** 10.1001/jamanetworkopen.2024.58984

**Published:** 2025-02-12

**Authors:** Jamie L. Hanson

**Affiliations:** 1 Learning, Research, and Development Center, University of Pittsburgh, Pittsburgh, Pennsylvania; 2 Department of Psychology, University of Pittsburgh, Pittsburgh, Pennsylvania

## Abstract

**Importance:**

Eviction and housing loss are pressing public health concerns. Understanding how caregivers’ stress about eviction or loss of housing is associated with specific childhood psychiatric issues across development is important.

**Objective:**

To examine associations between stress about eviction or loss of housing and caregiver-reported child depression, anxiety, attention-deficit/hyperactivity disorder (ADHD), and behavioral problems, while adjusting for sociodemographic factors.

**Design, Setting, and Participants:**

This cross-sectional study analyzed data from the 2022 National Survey of Children's Health, a nationally representative survey of caregivers and children in the US. Data were collected between July 2022 and January 2023.

**Exposure:**

Eviction or loss of housing.

**Main Outcomes and Measures:**

Caregiver-reported child depression, anxiety, ADHD, and behavioral problems. Generalized linear mixed models were used to test associations with stress about eviction or loss of housing.

**Results:**

From a sample of 54 103 families, 36 638 were included in the analytic sample (analytic sample mean [SD] age: 9.91 [4.62] years; sex assigned at birth, 18 901 male [52%]). In this study, stress about eviction or loss of housing was associated with depression in children (adjusted odds ratio [aOR], 1.10; 95% CI, 1.02-1.18). Stress about eviction or loss of housing was significantly associated with higher odds of reported anxiety in the base model (aOR, 1.26 (95% CI, 1.22-1.31) but not in the stringently adjusted model (aOR, 1.04; 95% CI, 1.00-1.08]). Associations were sometimes moderated by age, with stronger associations for younger children. Stress about eviction or loss of housing was not associated with ADHD or behavioral problems after adjustment.

**Conclusions and Relevance:**

This study found that stress about eviction or loss of housing was associated with increased odds of internalizing issues, such as depression and anxiety, among children. Prospective longitudinal research is still needed to fully understand these complex associations over time. These findings underscore the importance of policies and interventions to address housing instability and its mental health consequences for children.

## Key Points

**Question:**

What are the associations between caregiver stress about eviction or housing loss and children’s mental health outcomes across different ages?

**Findings:**

In this cross-sectional study of 36 638 children, stress about eviction or housing loss was associated with increased odds of depression, especially in younger children. No consistent associations were found with attention-deficit/hyperactivity disorder or behavioral problems after adjusting for different sociodemographic factors.

**Meaning:**

These findings suggest that stress about eviction or housing loss may differentially impact children’s mental health outcomes, particularly internalizing symptoms in younger children, and underscores the importance of housing stability interventions for children and their mental health.

## Teaser

This cross-sectional study examines the association of stress about eviction or the loss of housing with caregiver-reported depression, anxiety, attention-deficit/hyperactivity disorder, and behavioral problems for children aged 3 to 17 years in the US.

## Introduction

Eviction, foreclosures, and housing instability presents significant threats to the well-being of millions of people in the US each year.^1,2^ Millions of families face evictions, removal from their homes,^3^ or foreclosures.^4^ This is of incredible import for public health and public policy, as adults under threat of eviction or foreclosure report and evince multiple negative physical and mental health outcomes.^2,5,6^ Notably, eviction and foreclosures are not equally distributed across demographics—it disproportionately affects individuals from minoritized racial and ethnic groups, the economically marginalized, and families with children in their homes.^7^ Despite these alarming facts, there are many open questions associated with the impacts of eviction, foreclosures, and housing loss on child mental health, and deeper investigation is needed, especially regarding the etiology and occurrence of different psychiatric issues.

Research finds that eviction, foreclosures, and housing instability have significant health impacts on families and children, including premature birth, maternal depression, and parenting stress.^8,9,10,11^ Housing loss and eviction have been linked to decreased social support,^12^ food insecurity,^13,14^ increased conflict in the home,^15^ and harsh parenting.^16,17,18,19,20^ These challenges may cascade to significantly impact child development and mental health.^21,22,23,24^ For example, housing instability and experiencing multiple moves in childhood is associated with more externalizing problems^25^ or aggression, rule-breaking, and other disruptive behaviors that are expressed outwardly through actions.^26^ Similarly, household and residential changes in adolescence were associated with depression and internalizing problems, or issues that are inwardly directed, with distress in internal thoughts and feelings.^27^ Examined collectively, research generally suggests housing instability impacts child mental health, but further study is needed to understand the consequences of these experiences on child and adolescent functioning.

While existing research has provided insights into links between housing instability and child mental health, important gaps remain in fully understanding these associations. While individuals and families can be formally evicted via court order, informal evictions are much more common. Informal eviction is the most common reason (approximately 72%) for a forced move,^28^ as landlords may pressure, intimidate, or deceive a renter to leave without court proceedings.^29^ While foreclosures happen through court orders, there may be anticipatory shame, loss, and regret occurring as a borrower starts defaulting.^30,31,32^ As such, it is important to consider the psychological pressures and concerns associated with eviction or foreclosure to more fully understand these issues. Additionally, more work is needed to examine the association of eviction or foreclosure with mental health and children’s age. Eviction or loss of housing at specific points in development could have larger impacts on health; for example, younger children may be more impacted by stress and experiences as they are more dependent on caregivers and substantial brain development is occurring at this time.^33,34^ Age may moderate the impact of eviction or foreclosures given that different forms of psychopathology show differential onsets in childhood vs adolescence. Finally, there is substantial variability in how the impacts of eviction or foreclosures are isolated and what confounding variables are used in adjusting statistical models. Nearly all past projects include child demographics, while only some projects adjust for other developmental challenges common to families who face eviction (ie, food insecurity and low birth weight). Moving forward, it will be critical to think about psychological pressures and anxiety associated with eviction or loss of housing, while comprehensively adjusting statistical models and considering potential interactions with age.

To overcome these limitations, a national survey was used to test associations between caregivers’ stress about being evicted or housing loss and indicators of 4 forms of psychopathology—depression, anxiety, attention-deficit/hyperactivity disorder (ADHD), and behavioral or conduct problems. Given the wide-reaching impacts of eviction, I hypothesized that caregiver stress about eviction or loss of housing will be positively associated with risk for these 4 types of mental health problems and the association between caregiver stress about eviction or loss of housing and child mental health problems will be moderated by age, with greater associations seen in younger children.

## Methods

### Participants

This cross-sectional study followed the Strengthening the Reporting of Observational Studies in Epidemiology (STROBE) reporting guideline. Participants provided informed consent, but all data were publically shared without identifying information, and, therefore, did not meet the definition of human research and was exempt from institutional review board oversight at the University of Pittsburgh.

This study examined data from the 2022 National Survey of Children’s Health (NSCH), an ongoing population-based survey that collected responses from more than 50 000 caregivers in the United States.^35,36^ The NSCH is a high quality cross-sectional survey that was administered by the US Census Bureau and was collected across all 50 US states between July 2022 and January 2023. The survey collected data from 54 103 households (approximately 1061 per state). Parents or other knowledgeable adult caregivers answered questions about 1 specific child aged 0 to 17 years under their care. These caregivers completed a large battery of questions about their child, their demographics, their family circumstances, and multiple other domains.

### Measures

To measure stress about eviction or loss of housing, caregivers were asked 1 question about how often they were worried or stressed about being evicted, being foreclosed on or having their house condemned during the past year. Responses included always, usually, sometimes, rarely, and never. The coding of this item was reversed so that higher scores indicated greater concern and stress about these issues. Additionally, this study focused on caregiver reports of whether their children currently had or were previously told that their children had depression, anxiety, ADHD, or behavioral or conduct problems. These questions were recoded to be a binary indicator, with 1 indicating the child currently has the condition and 0 indicating does not have the condition or were ever told but does not currently have condition.

Regarding statistical covariates, models were adjusted for a basic set of demographic factors, including child sex, child age, child race or ethnicity, family structure, caregiver’s highest education level, and household poverty level based on federal guidelines and caregiver-reported family income (Table 1). Race and ethnicity was based on parental self-report data on the selected child’s Hispanic ethnicity and race, with imputation for missing cases. Given that eviction disproportionally affects individuals from minoritized racial and ethnic groups, these demographic factors were considered in our statistical models. Statistical models were more stringently adjusted for factors associated with, but independent of, eviction stress and included the number of places the child lived in the past year, if the child ever experienced homelessness, premature birth, low birth weight, poor maternal mental health, food insecurity during the past year, and exposure to adverse childhood experiences. These 2 sets of covariates were used to balance underadjustment and overadjustment biases. While it is critical to rule out potential confounding variables, overadjustment in social science can skew estimates away from the true total causal effect, prevent consistent estimation of associations, and introduce additional errors (eg, collider-stratification).^37,38^

**Table 1.** Demographic Characteristics of the Full Sample and Analytic Sample

Characteristic	Sample, No. (%)	
	Full (n = 54 103)	Analytic (n = 36 638)
Parent’s sex assigned at birth		
Male	16 478 (31)	10 345 (28)
Female	35 959 (69)	26 273 (72)
Missing	1666	20
Parent’s age, mean (SD), y	42.08 (9.50)	42.38 (8.20)
Missing	1692	74
Child’s sex assigned at birth		
Male	27 911 (52)	18 901 (52)
Female	26 192 (48)	17 737 (48)
Child’s age, mean (SD), y	8.60 (5.31)	9.91 (4.62)
Child’s race or ethnicity^a^		
Asian, non-Hispanic	3307 (6)	2042 (6)
Black, non-Hispanic	3292 (6)	1884 (5)
Hispanic	8370 (15)	5298 (14)
Multiracial, non-Hispanic	4401 (8)	2921 (8)
White, non-Hispanic	34 733 (64)	24 493 (67)
Household poverty status, % FPL		
0-99	6867 (13)	4044 (11)
100-199	8629 (16)	5447 (15)
200-399	15 674 (29)	10 735 (29)
400 or greater	22 933 (42)	16 412 (45)
Family structure		
Two parents, currently married	37 133 (71)	28 293 (77)
Two parents, not currently married	3084 (6)	2101 (6)
Single parent (mother or father)	10 441 (20)	6189 (17)
Grandparent household	1412 (3)	4 (0.01)
Other family type	483 (1)	51 (0.1)
Missing	1550 (2.8)	NA
Highest level of education in household		
Less than high school	1433 (3)	746 (2)
High school or GED	7046 (13)	4080 (11)
Some college or technical school	11 394 (21)	7549 (21)
College degree or higher	34 230 (63)	24 263 (66)

Abbreviations: FPL, federal poverty level; GED, general education development; NA, not applicable.

^a^ Multiracial, non-Hispanic includes: American Indian or Alaska Native Non-Hispanic, Native Hawaiian and Other Pacific Islander Non-Hispanic, and other multirace Non-Hispanic individuals.

### Statistical Analyses

Separate generalized linear mixed models with a logistic link function were used, where each form of psychopathology (a binary indicator) was entered as the dependent variable. Eviction stress and different covariates were entered as independent variables in base or stringently adjusted models, and we included a random factor for geographic location (50 US states and the District of Columbia). These multiple statistical adjustments were a way to address potential sources of bias. For parsimony, the interaction between stress about eviction or loss of housing and age was included in all models. The adjusted odds ratios (aORs) and 95% CIs were calculated for the independent variables of interest (eg, stress about eviction or loss of housing and stress about eviction or loss of housing × age). For significant interactions, the differences for the simple slopes of the association of stress about eviction or loss of housing were tested at different values of child age with respect to each form of psychopathology. Analyses were conducted using R version 4.4.0 (R Project for Statistical Computing)^39,40^ and only included participants with complete data for all variables across the 2 sets of covariates. Data were analyzed from April to November 2024. Statistical significance was set at *P* < .05, and all tests were 2-sided.

## Results

### Descriptive Statistics of the Sample

The full NSCH sample included 54 103 families, while the analytic sample included 36 638 families (analytic sample mean [SD] age, 9.91 [4.62] years; sex assigned at birth, 18 901 male (52%); 5298 Hispanic children [14%]; 24 493 non-Hispanic White children [67%]). Most participants came from families with 2 parents who were currently married in both the full sample (37 133 [71%]) and analytic sample (28 293 [77%]). The highest level of education in the household for most participants was a college degree or higher (full sample, 34 230 [63%]; analytic sample, 24 263 [66%]). Additional demographic statistics are noted in Table 1.

### Models Examining the Presence of Depression or Anxiety

These statistical models with a base set of covariates found several factors significantly associated with the presence of child depression, including family structure, race or ethnicity, caregiver education, poverty status, and sex assigned at birth (Table 2). Stress about eviction or loss of housing was significantly associated with higher odds of current depression (aOR, 1.35; 95% CI, 1.27 to 1.44; *z* = 9.348; *P* < .001). The interaction of stress about eviction or loss of housing × age was significant (*z* = −2.415; *P* = .02). The slope for stress about eviction or loss of housing was continually significant but varied at different ages (at lower levels of age −1 SD, aOR, 1.45; 95% CI, 1.30 to 1.62; *z* = 6.45; *P* < .001; at higher levels of age +1 SD: aOR, 1.26; 95% CI, 1.21 to 1.31; *z* = 11.37, *P* < .001). More stringent model adjustment attenuated some of these connections (Table 2). Stress about eviction or loss of housing was again significantly associated with higher odds of depression (aOR, 1.10; 95% CI, 1.02 to 1.18; *z* = 2.650; *P* = .008). The interaction of stress about eviction or loss of housing × age was significant (*z* = −2.505; *P* = .01). For these models, the slope for stress about eviction or loss of housing was significant in younger participants, but not for older participants (younger participants: aOR, 1.19; 95% CI, 1.05 to 1.34; *z* = 2.76; *P* = .006; older participants: aOR, 1.02; 95% CI, 0.97 to 1.06; *z* = 0.75; *P* = .45) (eAppendix 1 in Supplement 1).

**Table 2.** Associations Between Child Depression and Stress About Eviction or Loss of Housing

Characteristic	Base model		Stringent model	
	aOR (95% CI)^a^	*P* value	aOR (95% CI)^a^	*P* value
Race and ethnicity				
White, non-Hispanic	1 [Reference]	NA	1 [Reference]	NA
Hispanic	0.77 (0.66-0.89)	<.001	0.75 (0.64-0.87)	<.001
Black, non-Hispanic	0.35 (0.27-0.46)	<.001	0.38 (0.29-0.50)	<.001
Asian, non-Hispanic	0.30 (0.21-0.42)	<.001	0.38 (0.27-0.54)	<.001
Multiracial, non-Hispanic	1.00 (0.84-1.19)	.99	0.82 (0.68-0.98)	.03
Family structure				
Two parents, currently married	1 [Reference]	NA	1 [Reference]	NA
Two parents, not currently married	1.87 (1.53-2.28)	<.001	1.07 (0.86-1.32)	.56
Single parent (mother or father)	1.85 (1.64-2.09)	<.001	0.92 (0.80-1.05)	.19
Grandparent household	8.58 (0.79-92.7)	.08	5.85 (0.49-70.0)	.16
Other family type	5.57 (2.39-13.0)	<.001	2.19 (0.90-5.34)	.09
Highest level of education in household				
Less than high school	1 [Reference]	NA	1 [Reference]	NA
High school or GED	1.80 (1.24-2.62)	.002	1.56 (1.05-2.31)	.03
Some college or technical school	1.95 (1.35-2.82)	<.001	1.62 (1.10-2.39)	.01
College degree or higher	1.68 (1.16-2.44)	.006	1.67 (1.13-2.47)	.01
Household poverty status, % FPL				
0-99	1 [Reference]	NA	1 [Reference]	NA
100-199	0.95 (0.80-1.13)	.54	0.90 (0.75-1.08)	.25
200-399	0.89 (0.75-1.05)	.17	0.96 (0.81-1.15)	.66
400 or greater	0.79 (0.67-0.95)	.01	1.03 (0.85-1.24)	.77
Child’s sex assigned at birth				
Male	1 [Reference]		1 [Reference]	NA
Female	1.88 (1.71-2.08)	<.001	1.91 (1.73-2.12)	<.001
Child’s age, y	5.01 (4.59-5.46)	<.001	4.90 (4.47-5.37)	<.001
Eviction stress or concern	1.35 (1.27-1.44)	<.001	1.10 (1.02-1.18)	.008
Child’s age in years × eviction stress or concern	0.93 (0.88-0.99)	.01	0.93 (0.87-0.98)	.01
Places lived, No. of moves				
0-2	NA	NA	1 [Reference]	NA
3 or more	NA	NA	1.87 (1.42-2.46)	<.001
Ever homeless				
No	NA	NA	1 [Reference]	NA
Yes	NA	NA	1.42 (1.12-1.81)	.004
Mother’s mental health				
Excellent or very good	NA	NA	1 [Reference]	NA
Good	NA	NA	2.23 (1.98-2.50)	<.001
Fair or poor	NA	NA	3.30 (2.82-3.86)	<.001
Low birth weight				
No	NA	NA	1 [Reference	NA
Yes	NA	NA	1.02 (0.83-1.24)	.87
Born premature				
No	NA	NA	1 [Reference]	NA
Yes	NA	NA	1.09 (0.92-1.31)	.32
Food insecurity	NA	NA	1.16 (1.05-1.27)	.003
Adverse childhood experiences				
0	NA	NA	1 [Reference]	NA
1	NA	NA	1.83 (1.58-2.11)	<.001
≥2	NA	NA	4.85 (4.22-5.58)	<.001

Abbreviations: aOR, adjusted odds ratio; FPL, federal poverty level; GED, general education development; NA, not applicable.

^a^ Adjusted odds ratios from generalized linear mixed models examining associations between caregiver eviction stress and child depression.

Like depression, the base adjusted model found anxiety was associated with many sociodemographic factors (Table 3). Greater reported stress about eviction or loss of housing was significantly associated with higher odds of reported anxiety (aOR, 1.26; 95% CI, 1.22 to 1.31; *z* = 12.792; *P* < .001) in the base model. The interaction of stress about eviction or loss of housing × age was significant (z = −2.030; *P* = .04). The slope for stress about eviction or loss of housing was continually significant but varied at different levels of age (at lower levels of age −1 SD: aOR, 1.31; 95% CI, 1.23 to 1.39; *z* = 8.46; *P* < .001; at higher levels of age +1 SD: aOR, 1.22; 95% CI, 1.17 to 1.26; *z* = 11.69; *P* < .001). More stringent model adjustment attenuated associations with anxiety and sociodemographic factors (Table 3). In stringently adjusted models, stress about eviction or loss of housing was not significantly associated with higher odds of anxiety (aOR, 1.04; 95% CI, 1.00 to 1.08; *z* = 1.879; *P* = .06). The interaction of stress about eviction or loss of housing × age was not significant (*z* = −1.881; *P* = .06).

**Table 3.** Associations Between Child Anxiety and Stress About Eviction or Loss of Housing

Characteristic	Base model		Stringent model	
	aOR (95% CI)^a^	*P* value	aOR (95% CI)8^a^	*P* value
Child’s race and ethnicity				
White, non-Hispanic	1 [Reference]	NA	1 [Reference]	NA
Hispanic	0.73 (0.66-0.81)	<.001	0.72 (0.65-0.80)	<.001
Black, non-Hispanic	0.33 (0.27-0.40)	<.001	0.35 (0.29-0.42)	<.001
Asian, non-Hispanic	0.26 (0.21-0.32)	<.001	0.31 (0.24-0.38)	<.001
Multiracial, non-Hispanic	0.84 (0.74-0.95)	.005	0.73 (0.64-0.83)	<.001
Family structure				
Two parents, currently married	1 [Reference]	NA	1 [Reference]	NA
Two parents, not currently married	1.37 (1.19-1.58)	<.001	0.89 (0.77-1.03)	.12
Single parent (mother or father)	1.55 (1.42-1.69)	<.001	0.86 (0.78-0.95)	.002
Grandparent household	2.42 (0.24-24.6)	.45	1.89 (0.17-20.7)	.60
Other family type	2.91 (1.46-5.80)	.002	1.36 (0.67-2.80)	.39
Highest level of education in household				
<High school	1 [Reference]	NA	1 [Reference]	NA
High school or GED	1.58 (1.20-2.09)	.001	1.36 (1.02-1.82)	.04
Some college or technical school	1.89 (1.44-2.49)	<.001	1.60 (1.20-2.12)	.001
College degree or higher	2.03 (1.54-2.66)	<.001	2.01 (1.51-2.67)	<.001
Household poverty status, % FPL				
0-99	1 [Reference]	NA	1 [Reference]	NA
100-199	1.07 (0.95-1.22)	.28	1.04 (0.91-1.18)	.58
200-399	1.03 (0.91-1.16)	.63	1.09 (0.96-1.24)	.16
≥400	0.96 (0.85-1.09)	.55	1.19 (1.04-1.35)	.01
Child’s sex assigned at birth				
Male	1 [Reference]	NA	1 [Reference]	NA
Female	1.52 (1.43-1.62)	<.001	1.55 (1.45-1.66)	<.001
Child’s age, y	2.44 (2.34-2.54)	<.001	2.34 (2.24-2.44)	<.001
Eviction stress or concern	1.26 (1.22-1.31)	<.001	1.04 (1.00-1.08)	.06
Child’s age in years × eviction stress or concern	0.96 (0.93-1.00)	.04	0.97 (0.93-1.00)	.06
Places lived, last year				
0-2 times	NA	NA	1 [Reference]	NA
3 or more times	NA	NA	1.29 (1.05-1.60)	.02
Mother’s mental health				
Excellent or very good	NA	NA	1 [Reference]	NA
Good	NA	NA	1.88 (1.74-2.03)	<.001
Fair or poor	NA	NA	2.79 (2.49-3.12)	<.001
Ever homeless				
No	NA	NA	1 [Reference]	NA
Yes	NA	NA	1.29 (1.06-1.57)	.01
Low birth weight				
No	NA	NA	1 [Reference]	NA
Yes	NA	NA	1.00 (0.87-1.14)	>.99
Born premature				
No	NA	NA	1 [Reference]	NA
Yes	NA	NA	1.26 (1.12-1.41)	<.001
Food insecurity	NA	NA	1.22 (1.14-1.31)	<.001
Adverse childhood experiences				
0	NA	NA	1 [Reference]	NA
1	NA	NA	1.78 (1.62-1.94)	<.001
≥2	NA	NA	3.36 (3.05-3.70)	<.001

Abbreviations: aOR, adjusted odds ratio; FPL, federal poverty level; GED, general education development; NA, not applicable.

^a^ Adjusted ORs from generalized linear mixed models examining associations between caregiver eviction stress and child anxiety.

### Models Examining the Presence of ADHD or Behavioral Problems

The increased incidence of ADHD was associated with family structure, level of caregiver education, poverty status, and sex assigned at birth (Table 4). Stress about eviction or loss of housing was associated with greater rates of ADHD in the base model (aOR, 1.19; 95% CI, 1.15 to 1.23; *z* = 9.519; *P* < .001). The interaction of stress about eviction or loss of housing × age was not significant (*z* = −1.670; *P* = .09). When adjusting for a more stringent set of covariates, there were connections between ADHD and different covariates (eg, having low birthweight, childhood adversity) (Table 4). Notably, ADHD was not associated with stress about eviction or loss of housing (aOR, 1.03; 95% CI, 0.99 to 1.07; *z* = 1.441, *P* = .15) or the interaction of stress about eviction or loss of housing × age (*z* = −1.486; *P* = .14) in these models.

**Table 4.** Associations Between Child ADHD and Stress About Eviction or Loss of Housing

Characteristic	Base model		Stringent model	
	aOR (95% CI)^a^	*P* value	aOR (95% CI)^a^	*P* value
Child’s race or ethnicity				
Asian, non-Hispanic	0.31 (0.25-0.39)	<.001	0.36 (0.28-0.45)	<.001
Black, non-Hispanic	0.57 (0.48-0.67)	<.001	0.60 (0.50-0.70)	<.001
Hispanic	0.72 (0.64-0.80)	<.001	0.72 (0.64-0.80)	<.001
White, non-Hispanic	1 [Reference]		1 [Reference]	NA
Multiracial, non-Hispanic	0.92 (0.81-1.04)	.18	0.84 (0.73-0.95)	.006
Family structure				
Two parents, currently married	1 [Reference]	NA	1 [Reference]	NA
Two parents, not currently married	1.33 (1.16-1.54)	<.001	0.95 (0.82-1.10)	.48
Single parent (mother or father)	1.43 (1.30-1.56)	<.001	0.87 (0.79-0.97)	.008
Grandparent household	0 (0)	.95	0 (0)	.94
Other family type	5.09 (2.72-9.52)	<.001	2.57 (1.36-4.85)	.004
Highest level of education in household				
<High school	1 [Reference]	NA	1 [Reference]	NA
High school or GED	1.45 (1.10-1.90)	.008	1.28 (0.97-1.69)	.08
Some college or technical school	1.52 (1.16-1.98)	.002	1.30 (0.99-1.71)	.06
College degree or higher	1.33 (1.02-1.75)	.035	1.28 (0.97-1.69)	.08
Household poverty status, % FPL				
0-99	1 [Reference]	NA	1 [Reference]	NA
100-199	0.89 (0.78-1.01)	.07	0.87 (0.77-0.99)	.04
200-399	0.91 (0.81-1.03)	.14	0.95 (0.84-1.07)	.41
≥400	0.87 (0.77-0.99)	.04	1.02 (0.90-1.16)	.75
Child’s sex assigned at birth				
Male	1 [Reference]	NA	1 [Reference]	NA
Female	0.51 (0.47-0.54)	<.001	0.49 (0.46-0.53)	<.001
Child’s age, y	1.86 (1.78-1.94)	<.001	1.74 (1.67-1.82)	<.001
Eviction stress or concern	1.19 (1.15-1.23)	<.001	1.03 (0.99-1.07)	.15
Child’s age in years × eviction stress or concern	0.97 (0.94-1.01)	.01	0.97 (0.94-1.01)	.14
Places lived, last year				
0-2 times	NA	NA	1 [Reference]	NA
3 or more times	NA	NA	1.23 (0.99-1.53)	.07
Mother’s mental health				
Excellent or very good	NA	NA	1 [Reference]	NA
Good	NA	NA	1.55 (1.43-1.68)	<.001
Fair or poor	NA	NA	1.66 (1.47-1.87)	<.001
Ever homeless				
No	NA	NA	1 [Reference]	NA
Yes	NA	NA	1.51 (1.24-1.84)	<.001
Low birth weight				
No	NA	NA	1 [Reference]	NA
Yes	NA	NA	1.29 (1.13-1.47)	<.001
Born premature				
No	NA	NA	1 [Reference]	NA
Yes	NA	NA	1.18 (1.05-1.33)	.006
Food insecurity	NA	NA	1.14 (1.07-1.23)	<.001
Adverse childhood experiences				
0	NA	NA	1 [Reference]	NA
1	NA	NA	1.78 (1.63-1.95)	<.001
≥2	NA	NA	2.77 (2.51-3.07)	<.001

Abbreviations: ADHD, attention deficit/hyperactivity disorder; aOR, adjusted odds ratio; FPL, federal poverty line; GED, general education development; NA, not applicable.

^a^ aORs from generalized linear mixed models examining associations between caregiver eviction stress and child ADHD.

Finally, regarding behavioral or conduct problems, the increased incidence of this disorder was associated with race or ethnicity, family structure, and some indices of socioeconomic status in our base adjusted models (Table 5). Stress about eviction or loss of housing was associated with greater rates of behavioral or conduct problems in the base model (aOR, 1.22; 95% CI, 1.18 to 1.26; *z* = 11.151; *P* < .001). The interaction of stress about eviction or loss of housing × age was not significant (*z* = −0.175; *P* = .86). Stringent adjustment did not change most of these associations, but there were significant associations between behavioral or conduct problems and different covariates (eg, low birth weight, premature birth, food insecurity, childhood adversity) (Table 5). Of note, behavioral or conduct problems were not associated with stress about eviction or loss of housing (aOR, 1.00; 95% CI, 0.96 to 1.03, *z* = 0.122; *P* = .90) or the interaction of stress about eviction or loss of housing × age (*z* = 0.158; *P* = .87).

**Table 5.** Associations Between Child Behavioral Problems and Stress About Eviction or Loss of Housing

Characteristic	Base model		Stringent model	
	aOR (95% CI)^a^	*P* value	aOR (95% CI)^a^	*P* value
Child’s race or ethnicity				
Asian, non-Hispanic	0.41 (0.32-0.53)	<.001	0.52 (0.41-0.68)	<.001
Black, non-Hispanic	0.81 (0.68-0.96)	.02	0.92 (0.76-1.10)	.34
Hispanic	0.84 (0.75-0.95)	.006	0.86 (0.76-0.97)	.02
White, non-Hispanic	1 [Reference]	NA	1 [Reference]	NA
Multiracial, non-Hispanic	0.96 (0.83-1.11)	.55	0.85 (0.73-0.99)	.04
Family structure				
Two parents, currently married	1 [Reference]	NA	1 [Reference]	NA
Two parents, not currently married	1.30 (1.11-1.53)	.001	0.85 (0.72-1.01)	.07
Single parent (mother or father)	1.52 (1.37-1.69)	<.001	0.82 (0.73-0.92)	<.001
Grandparent household	2.55 (0.25-26.2)	.43	1.79 (0.13-24.0)	.66
Other family type	9.44 (5.24-17.0)	<.001	3.98 (2.14-7.42)	<.001
Highest level of education in household				
<High school	1 [Reference]	NA	1 [Reference]	NA
High school or GED	1.05 (0.79-1.39)	.74	0.89 (0.66-1.19)	.42
Some college or technical school	1.14 (0.86-1.50)	.36	0.93 (0.70-1.24)	.63
College degree or higher	0.99 (0.75-1.31)	>.99	0.95 (0.71-1.26)	.70
Household poverty status, % FPL				
0-99	1 [Reference]	NA	1 [Reference]	NA
100-199	0.94 (0.82-1.09)	.43	0.94 (0.81-1.09)	.41
200-399	0.86 (0.75-0.99)	.03	0.92 (0.80-1.07)	.28
≥400	0.78 (0.68-0.91)	.001	1.01 (0.86-1.17)	.93
Child’s sex assigned at birth				
Male	1 [Reference]	NA	1 [Reference]	NA
Female	0.41 (0.37-0.44)	<.001	0.39 (0.36-0.43)	<.001
Child’s age, y	1.06 (1.01-1.11)	.02	0.94 (0.90-0.99)	.02
Eviction stress/concern	1.22 (1.18-1.26)	<.001	1.00 (0.96-1.04)	.90
Child’s age in years × eviction stress or concern	1.00 (0.96-1.03)	.86	1.00 (0.97-1.04)	.87
Places lived, last year				
0-2 times	NA	NA	1 [Reference]	NA
3 or more times	NA	NA	1.50 (1.19-1.89)	<.001
Mother’s mental health				
Excellent or very good	NA	NA	1 [Reference]	NA
Good	NA	NA	1.91 (1.74-2.10)	<.001
Fair or poor	NA	NA	2.53 (2.23-2.87)	<.001
Ever homeless				
No	NA	NA	1 [Reference]	NA
Yes	NA	NA	1.67 (1.36-2.05)	<.001
Low birth weight				
No	NA	NA	1 [Reference]	NA
Yes	NA	NA	1.38 (1.19-1.61)	<.001
Born premature				
No	NA	NA	1 [Reference]	NA
Yes	NA	NA	1.14 (1.00-1.31)	.06
Food insecurity	NA	NA	1.18 (1.09-1.28)	<.001
Adverse childhood experiences				
0	NA	NA	1 [Reference]	NA
1	NA	NA	2.00 (1.79-2.24)	<.001
≥2	NA	NA	3.81 (3.38-4.29)	<.001

Abbreviations: aOR, adjusted odds ratio; FPL, federal poverty level; GED, general education development; NA, not applicable.

^a^ Adjusted odds ratios from generalized linear mixed models examining associations between caregiver eviction stress and child behavioral or conduct problems.

Associations between eviction stress and alternative controls are located in eAppendix 2 in Supplement 1. Recent American Academy of Pediatrics guidelines noted that certain mental health diagnoses were uncommon or not appropriate for children younger than 4 years^41^; thus, models were rerun with children younger than 4 years removed from the analysis, and these results are in eAppendix 3 in Supplement 1. eAppendix 4 in Supplement 1 examines the association between eviction stress and demographic characteristics. eAppendix 5 in Supplement 1 contains statistical models assessing the interaction of eviction and race. eAppendix 6 in Supplement 1 contains models ran for missing data. eAppendix 7 in Supplement 1 contains R markdown for all analyses in this study.

## Discussion

This cross-sectional study explored the association between caregivers’ stress about eviction or loss of housing and children’s mental health, namely depression, anxiety, ADHD, and behavioral or conduct problems. The strongest associations were between stress about eviction or loss of housing and 2 forms of internalizing psychopathology (ie, depression and anxiety). Stress about eviction or loss of housing was associated with a 10% to 35% increase in incidence of these types of disorders. Associations between stress about eviction or loss of housing, ADHD, and behavioral or conduct problems were less robust, with models adjusting for a base set of covariates finding associations. However, these results were not significant when estimates were adjusted with a more stringent set of potential confounders. Notable associations were observed between age and mental health and the interaction of stress about eviction or loss of housing. Specifically, these associations were strongest in younger samples and often became nonsignificant in older participants. Collectively, these findings may provide valuable information to researchers interested in the role of eviction in child development, particularly how this stressor may influence mental health.

These results fit with past findings that eviction, foreclosures, and housing loss may significantly impact development. These findings were connected with work noting increased depression in adolescents experiencing household and residential changes. However, consistent associations between stress about eviction or loss of housing and externalizing problems were not observed. This diverged from past work noting that housing instability and experiencing more than 3 moves in childhood was associated with aggression and other disruptive behaviors.^25^ This may be in part driven by our statistical modeling choices and the use of a stringent set of covariates. In both sets of models, stress about eviction or loss of housing was associated with depression; however, associations between stress about eviction or loss of housing and externalizing were only present in models adjusted for a base set of covariates and not in more stringently adjusted models. Externalizing problems were more associated with a child’s perinatal risk (ie, low birth weight; premature birth), but not all projects have controlled for these variables. The impact of eviction, foreclosures, and housing loss may be through more indirect developmental pathways, as it may contribute to perinatal risk, and this was associated with externalizing challenges. As such, future research is needed to comprehensively understand associations between stress about eviction or loss of housing and externalizing problems.

Our results were unable to parse potential differential impacts of eviction vs housing foreclosure, which should be considered in future work. Study participants were asked about multiple types of housing loss in 1 prompt (ie, eviction, foreclosure, or having a house condemned). To date, few studies have thoroughly investigated whether 1 type of housing loss may exert greater impacts on child and family well-being. Some scholars have suggested that foreclosure may be stressful for a more prolonged duration compared with eviction,^42^ while other researchers have lumped them under a similar category of disruptive events.^43^ Diamond et al^44^ found renters who were evicted due to landlords’ foreclosures had fewer adverse effects compared with those facing their own foreclosures. In this work, foreclosure on one’s own home was associated with later housing instability, reduced homeownership, financial distress, moves to lower-quality neighborhoods, and elevated divorce rates. However, further comparative research with direct comparison between eviction and foreclosure is needed to fully understand the impacts of these different types of housing loss.

This cohort was nationally representative and had a good deal of diversity in age, family structure, and socioeconomic status. Many past studies have only examined a smaller age range (eg, middle childhood), but this study had participants aged 3 to 17 years. With family structure, some past publications have used cohorts oversampling unmarried parents in large US cities.^45^ While appropriate, statistical weights must be used to have a nationally representative sample and make estimates generalizable to all families. Finally, it is important to note that while eviction or housing loss was concentrated in families at or below the federal poverty line,^7^ stress associated with these experiences still occurred for those with incomes above the poverty level (eAppendix 4 in Supplement 1). Examined collectively, this dataset allows for a more inclusive assessment of how the stresses associated with eviction or loss of housing are linked to child well-being.

### Limitations

This study has several limitations. First, this study is limited by its cross-sectional design, and longitudinal studies are needed to isolate the developmental impacts of eviction, which could be particularly informative for the interactions of age and stress about eviction or loss of housing. Stress about eviction or loss of housing was often no longer associated with the presence of different mental health disorders among older children in the cohort, which could index conflicting, developmental phenomena. For example, stress associated with housing loss that occurred early in life has outsized influences on development. Gaps in critical developmental skills may emerge early in childhood due to experiences before age 5 years.^46^ Alternatively, adolescents spend more time outside the home^47,48^ and the stress about loss of housing may have less opportunity to affect youth’s mental health. Prospectively following participants to assess how families deal with stress about eviction or loss of housing will be important to fully understand potential interactions between housing loss and development. Second, certain diagnoses are uncommon or not appropriate to diagnose in particularly young children.^41^ While some significant interactions of age and stress were found, the incidence of many of these disorders is low in younger children. Third, rich geographical information, such as neighborhood-related impacts of eviction, is not incorporated into the statistical models because the NSCH only provides information about which state a family resided in. Given varying state policies in eviction and housing protections, geographic differences could be important to understand. Finally, this study is limited by the use of caregiver self-reports of mental health challenges rather than independent measures or clinical interviews of psychopathology. Use of multiple sources (eg, teachers, clinicians) would have provided a comprehensive picture of the child’s functioning across different contexts (eg, home, school^49^), and may help guide treatment and intervention strategies.

## Conclusions

In this study, stress about eviction or loss of housing was associated with an increased incidence of mental health problems in childhood, particularly depression and anxiety. Given the profound long-term economic and social impacts of these problems,^50^ it will be critical for communities to think about lessening housing precarity and better supporting families facing eviction, foreclosure, or other housing loss. This could take many forms, including rental assistance, legal aid for tenants, eviction diversion programs, and expanded social safety nets.^51,52^ With millions facing evictions or foreclosures annually, those in public health and public policy must continue to push for expansion of these programs to reduce inequities and this key social determinant of child health.

## Notes

Supplement 1. **eAppendix 1.** Graphical Probing of Interaction Effects from Main Manuscript **eAppendix 2.** Relations Between Eviction Stress and Alternative Controls for Maternal Health **eAppendix 3.** Probing Associations Between Eviction Stress and Child Mental Health in Children Older Than Four Years of Age **eAppendix 4. **Relations Between Eviction Stress and Demographic Characteristics **eAppendix 5.** Probing Eviction Stress × Race Interactions **eAppendix 6. **Using Data Imputation for Missing Independent Variables **eAppendix 7.** R Markdowns

Supplement 2.
Data Sharing Statement
