# Supplementary material for: Stress About Eviction or Loss of Housing and Child Mental Health
Source: JAMA Netw Open. 2025 Feb 12;8(2):e2458984. doi: 10.1001/jamanetworkopen.2024.58984 (PMC11822551; doi:10.1001/jamanetworkopen.2024.58984)
Supplement: Supplement 2. — Data Sharing Statement [file jamanetwopen-e2458984-s002.pdf]

Data Sharing Statement

Hanson. Stress About Eviction or Loss of Housing and Child Mental Health. *JAMA Netw Open*. Published February 10, 2025. doi:10.1001/jamanetworkopen.2024.58984

Data
Data available: No

Additional Information
Explanation for why data not available: Data is publicly available at: https://www.census.gov/programs-surveys/nsch.html
